# Exploring ethical considerations for the use of biological and physiological markers in population-based surveys in less developed countries

**DOI:** 10.1186/1744-8603-1-16

**Published:** 2005-11-28

**Authors:** Gregory Pappas, Adnan A Hyder

**Affiliations:** 1 Chairman, Department of Community Health Science, Aga Khan University, 3500 Stadium Road, Karachi, Pakistan; 2 Assistant Professor, Dept. of International Health and Berman Bioethics Institute, Johns Hopkins University Bloomberg School of Public Health, 615 N. Wolfe Street, Baltimore, MD 21205, USA

**Keywords:** population based health surveys, ethical standards, less developed countries

## Abstract

**Background:**

The health information needs of developing countries increasingly include population-based estimates determined by biological and physiological measures. Collection of data on these biomarkers requires careful reassessment of ethical standards and procedures related to issues of safety, informed consent, reporting, and referral policies. This paper reviews the survey practices of health examination surveys that have been conducted in developed nations and discusses their application to similar types of surveys proposed for developing countries.

**Discussion:**

The paper contends that a unitary set of ethical principles should be followed for surveys around the world that precludes the danger of creating double standards (and implicitly lowers standards for work done in developing countries). Global ethical standards must, however, be interpreted in the context of the unique historical and cultural context of the country in which the work is being done. Factors that influence ethical considerations, such as the relationship between investigators in developed and developing countries are also discussed.

**Summary:**

The paper provides a set of conclusions reached through this discussion and recommendations for the ethical use of biomarkers in populations-based surveys in developing countries.

## Introduction

The national health information needs of developing countries are increasingly relying on the collection of biological and physiological measures [1-5]. The use of these biomarkers in population-based surveys has led to a call for a review of the ethical standards under which surveys are conducted in less-developed nations [6]. Controversies over clinical research conducted in developing countries have intensified the scrutiny of all research being conducted in these settings [7]. This debate has reaffirmed that the global human rights and medical ethical principles – including fidelity, truthfulness, confidentiality, autonomy, and beneficence, – must be carefully reviewed in the name of research, surveillance, monitoring, and evaluation [8-10]. Ethical issues in population-based surveys include informed safety, informed consent, confidentiality, and reporting findings of testing. Globalization of ethical principles must be interpreted within the particulars of national context in which the scientific study takes place.

Development of rapid, low cost, diagnostic kits and portable, robust technology has made possible a new generation of population-based health surveys [11]. While standards and safeguards for health interview surveys in developing countries have been in place for a number of years, the use of these new technologies adds a layer of new ethical issues to health surveys. The ethical issues raised by the collection of biomarkers in large-scale examination surveys, and standards for implementation have been developed over the past four decades in national population-based surveys done in developed countries [12]. The purpose of this paper is to review the current ethical practices and procedures used to guide survey work in the developed world, and discuss the implication of such practices for work in developing countries. The manuscript refers to written documents on these issues and draws upon health examination survey work of the authors in the United States and in less developed countries [3,13].

The article appears in three sections. The first section lays out the ethical principles that bind which are raised by health examination surveys, and reviews current practices. The use of these so called biomarkers has a long history in the National Health and Nutrition Examination Survey (NHANES) of the Centers for Disease Control and other major epidemiological studies (Framingham, Alameda county) [14,15]. These population-based health surveys have dealt with the following types of ethical issues: safety of the survey to subjects and workers in survey, obtaining appropriate informed consent, confidentiality of information collected in the survey, the reporting of findings of the health examination to the participant, the use of stored samples for research, and the provision of health care as part of a survey. While ethical principles may be global, implementation of those principles must be careful considered within local contexts in which the health examination survey takes place. In the second section these ethical issues will be addressed as they relate to conducting health examination surveys in less developed countries. This section emphasizes situations in which global standards have to be interpreted in a national or local context. While the spirit of the standard may be toward uniformity of procedures, implementation of surveys must consider many local conditions. Implementation of global standards in resource poor settings has created feedback and frequently challenges interpretation of those standards. The final section is a set of recommendations to be considered by national survey planners and donor agencies. While guidelines for health examination surveys have been developed over a long period in the US and Europe, those standards are under continued review as science and practice evolve. The globalization of standards creates a challenge for those standards and for feedback onto standards as practiced in the resource rich settings. Scientific advances and technological innovations will continue to require review of standards and procedures of ethical conduct in health examination surveys.

### I. Ethical Issues in Health Examination Surveys

This section reviews current practices being used in population-based health surveys in the US and other countries. Ethical practices for conducting health examinations surveys have developed over the past four decades in the United States and Europe related to safety, informed consent, confidentiality, the reporting of findings, and long-term sample storage in developed countries.

Safety issues in surveys consider consequences of collection of biological specimens and clinical testing for survey participants and for data collectors. Biomarkers that are minimally invasive are preferred (e.g., blood pressure measurement, venous blood draw or urine collection). Potentially dangerous tests (high intensity x-rays) have usually been avoided. The use of minimally invasive, as opposed to potentially dangerous procedures has important implications to the risk/benefit ratio of the survey. Safety standards for specimen collectors typically follow clinical standards, and universal precautions for laboratory work have been adopted for field use.

Informed consent in health examination surveys has been most frequently obtained in writing in developed nations. Concerns about obtaining informed consent in special populations have lead to accommodations or modified procedures. In special populations – those with low levels of literacy, different cultural traditions, a context of inequities, or where there is lack of health services, – informed consent requires special consideration. An empirical literature has developed around the informed consent process that raises concern about what participants understand or remember about the consent process [16]. Judgment about the appropriate level of effort to achieve informed consent hinges on the risks and benefits of the survey. For example, clearly worded, plain language, consent has been deemed acceptable in surveys that convey minimal to low risk and additional efforts to ensure complete comprehension of the purpose of the survey are not routinely made.

Maintaining the confidentiality of information from survey participants is an important responsibility including specific components, such as privacy during interviews (particularly around sensitive issues) and prevention of inadvertent disclosure of identity, in analysis and data release products. Concerns about confidentiality are increased with biomarkers because they have the potential for disclosing sensitive health information (e.g., disease status, diabetes, HIV) leading to the misuse of this information to profile individuals for social and health (insurance) purposes. Legal standards to ensure confidentiality have been adopted by governmental statistical agencies [17].

The report of findings of individual laboratory or physiological testing back to survey participants has historically been done in three categories: standard reporting, routine referral, and urgent referral. For example, normal levels of blood pressure are reported to a subject in a standard way. Elevated levels are reported and recommendations or follow-up are made (referrals). Very high levels require urgent referral; recommendations for action are made for urgent action.

When investigators know, or can know, the identity of the tested individual, they are required to report back to the survey participant unless the consent process specified otherwise. A person can consent that a result will not be reported. Consent for not reporting has been considered appropriate when the result of the test has little or poorly understood significance to an individual's health. An example of such a test was the assay for homocystene that was done as part of the NHANES III. The laboratory results for homocystene (that has been under study as a risk factor for cardiovascular diseases) were not reported to participants or their physicians because its physiological or clinical significance was not known. Disease markers that have specific and potentially important health consequences must always be reported to the individual if the investigators can identify the individual and connect the results of the test to that individual. An alternative that has been used to address this issue is to "anonomize" the samples by stripping the identifiers from the sample in a way that makes it impossible to identify the survey participant. In either case, the consent statement should make clear whether the results will or will not be reported back to participants.

Implementation of reporting also depends on a variety of issues in a survey setting. Laboratory analyses that are not performed in the field can not therefore be reported to survey subjects at the time of the survey. Reporting at a time after the initial field work can be a major challenge for large scale surveys. It may also be desirable to share the test results of an individual with a physician designated by the survey participant. Reporting of results in a form that is understandable to survey participants needs to be given high priority when implemented. Reports of survey findings to respondents may also include recommendations of appropriate actions to be taken based on the findings.

Long-term storage of biological specimens collected in population-based surveys has also been common and raises other ethical concerns. Intention to store samples should be included in the consent process. Frequently, biological samples are stored without specific plans for analysis and because testing may take place in the remote future (years, even decades in the future) it cannot be anticipated what tests it will be possible to conduct. It may not be feasible to guarantee that the investigators could reliably contact the person from whom the sample was obtained.

Long term storage also has created the possibility for genetic testing of samples collected in health examination surveys. Standards are still involving the United States and Europe [2]. The U.S. National Center for Health Statistics/CDC/DHHS has approved of six protocols for genetic testing of specimens collected as part of national populations based household surveys (Health and Nutrition Examination Survey, NHANES, III) [18]. Consent in that survey included broad permission for doing unplanned future testing. Genetic tests had not been planned in the original survey and was not specifically included in the consent. With the extensive involvement of the IRB genetic testing has begun under protocols that are considered exploratory. White cells were used to create over 8000 cell lines for future genetic testing. The samples were "anonomized" so that no investigator, including those inside the government can link the sample to the identity of a sample person. The on-going involvement of the NCHS IRB will hopefully lead to creation of guidelines that may be useful to other research settings.

### II. Ethical Issues in Examination Surveys in Developing Countries

Universal standards may be the preferable approach to guiding ethical conduct in surveys. Application of universal standards for ethical behavior in survey work in diverse settings, however, requires careful consideration. Implementation of universal standards must consider cultural settings, the health care delivery system of the country, national legal frameworks, and the context of relationships of investigators. This section will discuss ethical issues raised by large-scale surveys in less developed countries that take biological and physiological measures.

#### Safety

International safety guidelines for the collection of biological and physiological measurements in surveys are the norm for surveys conducted in less developed countries. However, as with clinical practice, achieving these safety guidelines is frequently difficult. Financial constraints in less developed countries often lead to clinical practices that do not follow universal precautions. Lack of appropriate equipment and lack of training may leads to clinical practices that put patients and health care workers at risk. The clinical realities and experience of those who are recruited to work in a health survey has consequences for the implementation of safety guidelines. Safety guidelines may be difficult to enforce in surveys with data collectors drawn from clinical settings in which universal precautions are not observed. Conflicts between international advisors and country implementation staff may surface around expenditures on equipment that is considered unnecessary (latex gloves, disposal facilities). Clear contractual agreements and supervision concerning safety precautions are critical to the maintenance of safety standards.

#### Informed Consent

Many aspects of consent in examination surveys are similar to standards used in interview surveys in less developed countries. Standards for informed consent must be adapted to cultural circumstances of the country in which the surveys are conducted [19]. Verbal documented consent is routinely used in settings of low literacy. In addition, cultural hierarchies may demand that approval is obtained from the authority figures in the local context (e.g., village elders, head of family). Translation problems must be addressed; often many of the words needed to explain the survey or a procedure do not translate into local languages. The language of consent forms needs to address the specific settings [20].

While coercion or inappropriate inducement is considered a violation of consent, financial incentives in developed country settings are a common practice [21]. Financial incentives are seen as a benefit to off-set lost wages or the opportunity cost of time spent and effort required for participation. The poverty in many countries in which surveys are conducted, means that even the smallest financial incentive may raise concern about undue inducement to participate. Levels of financial incentives for participation that is optimal without being coercive is an empirical issue and must be established for each setting.

Much of the contemporary controversy concerning ethics of research done in less developed countries comes out of clinically oriented research or drug efficacy trials. A distinction between experimental medical research and population-based health surveys helps clarify this discussion of ethical guidelines. The risk inherent in clinical research imposes a responsibility on the investigator for the well being of human subjects. In contrast, surveys collect data that describe health conditions of a population, or monitor and evaluate population-based programs, and usually are of minimal to low risk to human subjects. The risk of population-based surveys is typically related to the burden of questions, time, and sample of body fluid or tissue. The difference between clinical studies and survey research must be made clear to institutional review boards. Including survey expertise on the membership of an institutional review board is a good way to inform these boards of the particular issues raised by survey research.

While these ethical principles are well established, specific procedures (e.g., wordings, verbal versus written, assurance of comprehension) are very much tied to the specific context of the research (complexity of the issues that require consent, risks and benefits). Globally agreed upon principles do not simplify the process of establishing informed consent, and a sometimes lengthy consultation is needed. However, complexities of informed consent are common to resource rich and resource poor settings.

#### Confidentiality

The practical considerations related to confidentiality in highly developed countries and the less developed countries may differ due to legal requirements, cultural issues, and logistical constraints of the field research. The primary concern in the US and other developed countries is that survey data is not used to disclose the identity of an individual. Linkage of data systems and profiling (by health care providers or insurers) presents concerns about confidentiality in some settings. These sorts of concerns are usually of low priority in less developed countries because such risks are usually minimal or non-existent. In many less developed countries people may not have specific addresses or standardized naming systems. These conditions make it difficult to locate persons after the initial contact of a survey. In a practical sense this reality creates confidentiality and represents a safe guard. In addition, stripping data of unique identifiers and data control procedures are practices that help maintain confidentiality and should be adopted universally [22].

Perhaps more critical in developing countries is privacy in the field setting. Inadvertent disclosure of medical information to family or neighbors during the data collection process in the fields setting may be the major privacy issues raised by field surveys in less developed countries. Surveys conducted in less developed countries, particularly in rural settings and in household surveys, make privacy in data collection a problem. Lack of privacy in household survey settings has consequences on data quality and potential breaches of confidentiality. Communal ways of living, large families, joint households and other elements mean that people congregate quickly and individual interviews are challenging. Training of field staff, careful supervision, and quality control in field work related to privacy standards, are essential if privacy is to be maintained in field settings.

#### Reporting Results

The reporting of findings of individual biological and physical measurements to survey participants is determined, in part, by the nature of the parameter and the clinical meaning (significance) of the parameter which have universal implication. The social meaning of the disease or condition, the social context in which the report is made and the nature of the health care delivery system, which differ in countries, determine the appropriate manner in which the reporting is done in less developed countries. Health examination surveys in less developed countries have followed U.S. formats for reporting of test results by reporting three categories: standard reporting, routine referral, and urgent referral.

The social meanings of a report of findings in a particular country context may influence the way that a report of a finding is provided. For example leprosy or epilepsy in stigmatized some cultures require additional consideration in reporting. Privacy in reporting would need to be heightened in these contexts.

The policy for reporting the findings of biological and physiological measures to participants of population-based surveys must also consider where the sample testing actually takes place and how the reporting take place. Measures taken in the field for which results are immediately available (blood pressure, rapid testing of blood) can be reported as a routine part of field work. Specimens collected in the field and tested in central laboratory require other reporting procedures. Telephoning or mailing reports of findings is the preferable policy but this may not be feasible in many less developed countries. Long distances, difficulty with travel, and difficulties with the re-identification of sample persons make reporting of centralized testing very difficult.

An option exists to resolve the issues of difficulties raised by having to return to households with test results done after the initial visit. Obtaining consent not to report the particular finding has been acceptable solution. In conditions for which treatments are not available, reporting may not be useful to the sample person (e.g., HSV2) and may not be necessary. Nonetheless consent for not reporting has been recommended. Ethical review committees must weigh the risks and benefits of these types of trade-offs in surveys. The risks and benefits of participating in the survey must be considered with a clear definition of instances under which not reporting findings would be considered appropriate.

The nature of the health care delivery system (that is, the level of its development) is also critical to the report of findings. Reporting a finding and recommending an action that is not a realistic possibility for the patient is a usual challenge that policy for report of findings must face. Referral to the existing standard of care in the country is the usual policy that has been proposed. More on the issue of the responsibilities of survey researchers to provide services is discussed in the next section

#### Service and the Provision of Care

The finding of abnormal results of a biological or physiological measure in a population-based survey may necessitate referral. In less developed countries the policy for referral must consider the types of services that are available to the survey population. A referral to a poorly functioning health care delivery system is problematic. The notion that the prevailing standard of care in the country is a sufficient referral has been challenged by some [23]. These sorts of challenges have been faced in the context of clinical research in which investigators from countries with higher levels of services have offered lower levels of care. In clinical research, investigators may have a relationship with the study populations over a period of years and subject the population to circumstances (drugs) that are not risk free. The referral to prevailing standards of care in the country may not be considered adequate. However, since population-based surveys make only transient contact with the participants (that is a clinical relationship is not established or intended) and because risk is generally low, relying on the prevailing standard of health care in the areas may be appropriate.

Does the absence of adequate medical services imply that tests in surveys should not be performed? The question might be reframed, "To what extent is a report of a disease or condition considered to be a benefit when treatment is not immediately available?" Risks of knowledge, in and of itself, may be considered minimal. It can be stated that the primary purpose of data collected in a national survey is not to provide benefit at the individual level, but to collect information to benefit the whole population in the long term. Thus, a discussion of the benefits from a survey needs to balance the immediate benefit to participants and to the nation.

Ethical considerations about beneficence are often mixed with humanitarian concerns to provide health care services to those in need. Historically in the United States the consideration of risks and benefits of most surveys has not lead to recommends that require that health care be provided to survey participants. The health care needs of participants and their communities fuels considerations to use survey work as a vehicle to provide care. The doctors or nurses on the survey team may be the only health care professional that is actually available in a resource poor setting. However, building a transient health care provision system onto a survey can not take the place of national health care system development. In addition, ethical considerations about coercion or inducements must be considered. Survey participation should not be perceived by the participants as a criterion for receiving services.

#### Special considerations for HIV testing

HIV testing in national population-based surveys in less developed countries has been successfully completed in a number of less developed countries [4,5,24]. The inclusion of HIV as a biomarker requires special consideration because of the stigma attached to the disease and the life threatening nature of the disease. Disclosure of HIV status is a life and death issue in some countries making the standards for confidentiality in such surveys very high. Confidentiality must be maintained in the field (protection of disclosure to family and neighbors in communities) and in data sets (protection of survey participants from use of the data set to identify HIV positive individuals). The burden of maintaining confidentiality among field staff members in the context of rapid testing in the field is a real concern. In a number of research protocols, it would be important to debate the obligation to report the finding if the researchers can know the HIV status of a person in a study.

De-linked protocols ensure the confidentiality of the data, but disappoint advocates of voluntary counseling and testing (VCT) who look at reporting results to survey participants as a service. Indeed, HIV test results (received voluntarily) can be considered as a public health good and as a benefit to the individual. Reconciliation of needs for confidentiality and service has been found in the provision of VCT services in conjunction with surveys that collect de-linked data. This necessitates repeat testing of those who want to know their HIV status.

### III. Discussion and recommendations

This discussion of the ethics related to the incorporation of biomarkers into population-based surveys is a starting point for agencies conducting health surveys in less developed countries and those providing technical assistance and funding. Institutional responses to ethical conduct of science in less developed countries have been emphasized here and this institutional capacity should be considered integral to the capacity building in health that is supported by many international donor agencies. Developing countries must have their own sustainable institutional frameworks to discuss, develop, and implement ethical protections which are morally sound and nationally relevant [25]. International harmonization of ethical principles and procedures as they relate to population-based surveys using biomarkers need to be established. A set of standards for ethical practice may be preferable to the development of different standards for developed and less developed settings. Current practices in use may be appropriate but need to be explicitly discussed and debated for adoption in less developed settings. While implementation of surveys will differ in various countries, the principles underlying their conduct should not differ. Ethical issues raised by population-based surveys are distinct from those raised by clinical research. Defining the benefit of survey work to participants needs to be made explicit but the mission of a survey should not be confused with a clinical mission. At the same time, the knowledge or specific information a sample person learns about their health status should not be considered an inducement for survey participation.

Development of capacity for ethical review of survey research in less developed countries should be a part of technical assistance from high income countries. While ethical principles may be global, procedures must be adapted to local conditions, and local IRBs must be able to ensure that ethical principles are translated into implementation plans. Survey activities would be well served by the development of more sophisticated Institutional Review Boards in host countries that undertake survey work. Development agencies and their contractors may wish to invest in this development, and capacity building in the health sector should formally include work in this area. The composition of IRBs should also include those who understand the technical aspects of survey work, as the particulars of research are essential in the appropriate evaluation of ethical procedures and standards.

Population-based surveys frequently have political consequences that must be anticipated by donors and host countries. While these considerations are important, they should not become confused with the ethical standards that frame survey design. The institutional integrity of IRBs in donor and in host-recipient countries is essential. The power differential between donor and host institutions must be recognized when institutional rules are established. A practical recommendation is for the cost of the ethical review to be borne by the research partner from the high income country as part of the technical assistance offered in a particular research project. Studies funded by rich countries should fund ethical review of surveys in poor countries in such a way that level of functioning and autonomy of local IRBs (Institutional Review Boards) can be ensured.

International donor agencies should facilitate collection of research experience and dissemination of such experience. An exploration of such practices can be done to establish contemporary practices. The National Center for Health Statistics of the Centers for Disease Control and Prevention (CDC) has conducted the National Health and Nutrition Examination Survey (NHANES) in the United States for over 30 years. The written policies and procedures of the NHANES  represent the standard for the use of health assessments in population-based surveys in the United States.

Because many biological specimens can be used for genetic testing, specimens being collected in less developed countries will draw great interest. One practical recommendation to address this inevitability is to follow the approach that is being used in the United States that has allowed provisional use of samples for genetic testing in an exploratory setting under on-going scrutiny of an IRB. This proposed exploratory study of ethical standards should be well supervised and well staffed by IRBs in both the donor country and in the host country. A well conducted study will maximize the learning concerning ethical standards for genetic testing and safe guard the rights of the sample persons. IRBs should require intimate involvement in such exploratory work with frequent reporting back to evaluate the practical issues raised by genetic testing. Who owns genetic materials once it is collected is a unique issue that must be addressed. Experience with genetic testing in less developed countries may have implication for a discussion that is increasingly global in nature.
